# Optical coherence tomography angiography in thyroid associated ophthalmopathy: a systematic review

**DOI:** 10.1186/s12886-024-03569-5

**Published:** 2024-07-22

**Authors:** Mohammad Taher Rajabi, Reza Sadeghi, Mohammad Reza Abdol Homayuni, Saharnaz Pezeshgi, Seyedeh Simindokht Hosseini, Mohammad Bagher Rajabi, Sepideh Poshtdar

**Affiliations:** 1 Eye Research Center, Farabi Eye Hospital, Tehran University of Medical Sciences, Zip Code: 1336616351, Tehran, Iran; 2 School of Medicine, Tehran University of Medical Sciences, Tehran, Iran; 3 Students’ Scientific Research Center, Tehran University of Medical Sciences, Tehran, Iran

**Keywords:** Thyroid-associated ophthalmopathy, Thyroid-associated ophthalmopathy, Graves' orbitopathy, Optical coherence tomography angiography

## Abstract

**Purpose:**

To evaluate the evidence for alterations of blood flow, vascular and perfusion densities in the choroid, macula, peripapillary region, and the area surrounding the optic nerve head (ONH) in patients with thyroid-associated ophthalmopathy (TAO) based on changes of OCTA parameters.

**Methods:**

A systematic review of Pubmed, Google Scholar, Scopus, WOS, Cochrane, and Embase databases, including quality assessment of published studies, investigating the alterations of OCTA parameters in TAO patients was conducted. The outcomes of interest comprised changes of perfusion and vascular densities in radial peripapillary capillary (RPC), ONH, superficial and deep retinal layers (SRL and DRL), choriocapillaris (CC) flow, and the extent of the foveal avascular zone (FAZ).

**Results:**

From the total of 1253 articles obtained from the databases, the pool of papers was narrowed down to studies published until March 20th, 2024. Lastly, 42 studies were taken into consideration which contained the data regarding the alterations of OCTA parameters including choriocapillary vascular flow, vascular and perfusion densities of retinal microvasculature, SRL, and DRL, changes in macular all grid sessions, changes of foveal, perifoveal and parafoveal densities, macular whole image vessel density (m-wiVD) and FAZ, in addition to alterations of ONH and RPC whole image vessel densities (onh-wiVD and rpc-wiVD) among TAO patients. The correlation of these parameters with visual field-associated parameters, such as Best-corrected visual acuity (BCVA), Visual field mean defect (VF-MD), axial length (AL), P100 amplitude, and latency, was also evaluated among TAO patients.

**Conclusion:**

The application of OCTA has proven helpful in distinguishing active and inactive TAO patients, as well as differentiation of patients with or without DON, indicating the potential promising role of some OCTA measures for early detection of TAO with high sensitivity and specificity in addition to preventing the irreversible outcomes of TAO. OCTA assessments have also been applied to evaluate the effectiveness of TAO treatment approaches, including systemic corticosteroid therapy and surgical decompression.

**Supplementary Information:**

The online version contains supplementary material available at 10.1186/s12886-024-03569-5.

## Introduction

Thyroid eye disease (TED), also known as thyroid-associated ophthalmopathy (TAO) or Graves' orbitopathy (GO), is characterized as a chronic autoimmune inflammation of orbital and periorbital tissues causing expansion of orbital fat, extraocular muscle (EOM) inflammation, eyelid retraction and compression of optic nerve (ON) in some cases. TAO, being closely related to Grave's hyperthyroidism, can manifest itself through various clinical presentations, including diplopia and visual impairment; also, it is the most common cause of proptosis in adults [1–4]. One of the most critical aspects of TAO is the changes in orbital blood flow and hemodynamics, which occur during the disease and orbital inflammation. Subsequent to pathologic consequences of hyperthyroidism, such as orbital inflammation, elevated blood pressure, and intraocular pressure (IOP), the orbital flow might be affected, leading to hypoxia and damage to ON [5].

Orbital cavity hemodynamics alterations, such as congestion of orbital veins and decline of superior ophthalmic vein (SOV) flow rate, have been confirmed in TAO patients. Venous obstruction in TAO is mainly caused by external compression, and it can lead to periorbital swelling, proptosis, and chemosis. Orbital congestion-induced increase of episcleral venous pressure, elevated retrobulbar pressure, and EOM contraction-induced globe restriction can increase IOP levels in TAO patients [6]. TAO patients also have been confirmed to have remarkably different bulbar conjunctival microvasculature morphology and hemodynamics [7].

The blood flow elevation was more prominent in the choroid than the retina following the treatment of active TAO, suggesting choroidal flow monitoring as an essential means of orbital pressure elevation evaluation in TAO patients. Furthermore, dysthyroid optic neuropathy (DON) patients have markedly lower orbital blood flow, higher orbital vessel resistivity index, and velocity compared to non-DON (NDON) patients. The controversy over the decrease or increase of ocular perfusion in TAO may be due to variations of systemic conditions, IOP, inflammatory orbit status, and differences in scanning area or OCTA type and model [5, 8].

As mentioned before, timely diagnosis of TAO is a crucial factor in the treatment outcome and prevention of irreversible damage to the optic nerve and disruption of visual function [9]. OCTA, a state-of-the-art, non-invasive volumetric imaging technique, has proven effective in the detailed evaluation of retinal vasculature, optic disc perfusion, and choroidal blood vessel flow alterations in a few seconds [10]. OCTA is a possible candidate for replacement of dye-based Fluorescein angiography (FA) and Indocyanine green angiography (ICGA) angiographic methods; these techniques, however effective, are considered invasive and eye movements and optical phenomena could confound their results in the process of image acquisition; altogether suggesting a paradigm shift from dye-based angiographic methods towards the application of OCTA as a first line means of diagnosis due to significant agreement between these imaging methods, which was confirmed via deep learning approaches [10, 11].

In recent years, several studies have evaluated the retinal and choroidal changes in the process of TAO using OCTA imaging; therefore, here we performed a systematic review of the literature in order to assess the alterations of OCTA parameters in the process of TAO, relativity of these parameters to disease activity and severity, diagnostic and prognostic values of these parameters, and their relevance to changes of parameters associated with visual function.

## Literature search method

To perform a thorough literature review, we searched the Pubmed, Google Scholar, Scopus, Embase, Web Of Science,and Cochrane databases using the phrases “thyroid ophthalmopathy”, “TAO”, “thyroid associated ophthalmopathy”, “grave's orbitopathy”, “grave's ophthalmopathy”, “thyroid eye disease”, “TED”, “Optical coherence tomography angiography”, “OCT-A”, “OCT angiography”, “vascular density”, “peripheral density”, “choroidal vascularity index”, “retinal microvasculature”, “choroidal microvasculature”, “retinal microcirculation”, and “choroidal microcirculation”. The search was performed on March 20th, 2024. No time restriction was proposed. Two authors SP(Sepideh Poshtdar) and MRAH independently screened titles and abstracts and categorised them into one of three selections—“include”, “exclude”, and “maybe”. Disagreements between reviewers were resolved with discussion and arbitrated by senior reviewers (RS and MTR) where there was a failure to reach consensus Case reports, irrelevant articles, articles in any languages except English, and articles without available full text and insufficient data in the abstract were excluded.

## Results

**Fig. 1:**
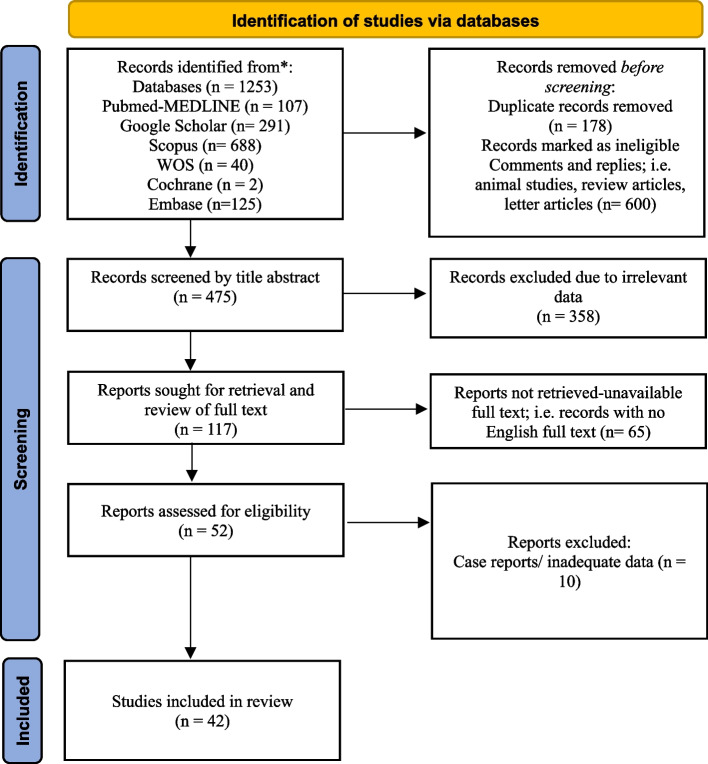
PRISMA flow diagram of literature review process

A total of 1253 articles were included. Exclusion of duplicates yielded 1075 unique articles, 600 of which were found to be ineligible. Title abstract screening yielded 358 irrelevant articles. The remaining 117 articles were sought for retrieval, however 65 articles were not retrived due to unavailable full text or no English full text. Full-text screening was undertaken for the remaining 52 articles. During the full-text screening process, 10 studies were excluded. The remaining 42 results were selected for critical appraisal and included in the synthesis of our results (Fig. 1).

### Risk factors and pathophysiology of TAO

Regarding the risk factors of TAO, in addition to smoking being the most relevant one, there exist other risk factors as well, including radioactive Iodine treatment (RAI), genetic factors, gender (more severe disease advancement in men despite higher prevalence in women) and racial components (e.g., increased possibility of TAO development in Asians compared to Caucasians) [12].

Despite the remaining ambiguities in understanding the pathophysiology of TAO, the role of thyroid stimulating hormone receptor (TSHR) and the insulin-like growth factor-1 receptor (IGF-1R), is well stablished, as they contribute to initiation of a complex immune cascade and inflammatory processes involved in the disease advancement [13, 14]. During the active phase of TAO, due to inflammatory changes, consequent orbital space compression, and elevated IOP, orbital vascular structures may become compressed, and hemodynamics may be influenced, representing increased venous pressure, retro blood flow, lowered flow, and inclined choroidal vascular resistance. Venous congestion acts as a key component of orbitopathy during active phase, which can be associated with clinical signs and symptoms such as proptosis, chemosis, EOM restriction and periorbital swelling [15–17]. Moreover, retrobulbar pressure (RBP) is significantly increased in TAO and it can be responsible for dysfunction of ON, in addition to swelling of EOM. Furthermore, DON patients were confirmed to have enlarged SOV in CT scan imaging, suggesting it as a potential associated factor for optic neuropathy [15, 18, 19].

### Clinical scoring and treatment of TAO

A comprehensive evaluation of TAO is highly necessary to select a viable treatment plan based on disease severity and activity. Rundle's curve is being applied for distinguishing the active and inactive phases of the disease. The active phase typically lasting from 6–18 months is accompanied by acute inflammation which responds well to irradiation and corticosteroid therapy, through the subsidence of this phase, the disease enters into a chronic fibrosis-associated phase in which corrective surgeries could be performed to reverse complications of TAO such as proptosis and eyelid deformities [20, 21].

#### TAO activity

Although there are other grading systems, such as VISA (Vision, Inflammation, Strabismus, Appearance) score and MRI sequencing for evaluation of TAO, the clinical activity score (CAS), despite its limitations, is yet considered the best scoring system for TAO activity. CAS is composed of seven components (illustrated in Supplement file, Table 1), and if present, each gains one score, a CAS of 3 or higher is considered as active TAO, and a CAS of lower than 3 is regarded as inactive TAO [22].

#### TAO severity

As for the disease severity, there are multiple scoring systems such as the European Group on Graves’ Orbitopathy (EUGOGO) qualitative classification, in addition to VISA, NOSPECS, and total eye score quantitative systems. According to EUGOGO, TAO patients are classified into groups of mild, moderate-to-severe, and sight-threatening TAO (illustrated in Supplement file, Table 2). Moreover, the NOSPECS scoring system is a 0–6 grading system as follows: 0: No signs and symptoms of TAO, 1: Only signs of TAO, 2: involvement of orbital soft tissue, 3: Presence of proptosis, 4: Involvement of EOM, 5: Corneal involvement, 6: Vision loss [23].

#### Treatment modalities applied for TAO

Due to their immunomodulatory characteristics, corticosteroids have been the first-line treatment option of TAO since the 1960s. In moderate to severe active TAO, intravenous glucocorticoids are considered a viable treatment option for silencing the disease and providing the opportunity for rehabilitative surgery in the inactive phase; however, in cases of sight-threatening TAO, urgent intervention is required if there is no improvement following two weeks of IV glucocorticoid therapy. In addition to corticosteroids, the application of monoclonal antibodies (e.g., Rituximab, Tocilizumab, and Teprotumumab) has been reported in the treatment of TAO in clinical trials. Orbital radiotherapy being a well-known treatment for TAO, is a beneficial adjuvant to steroid therapy in cases of active moderate to severe disease. Almost 20% of TAO patients require surgery despite proper medical treatment. Poor corticosteroid response in sight-threatening cases, demands immediate orbital decompression [24]. Decompression orbitomy surgery can lead to attenuation of IOP, and it's effective in patients with Compressive optic neuropathy (CON), severe proptosis, and exposure keratopathy via resolving the discrepancy of elevated volume of EOM and inflexible bony structures [18].

### Diagnosis of TAO; the application of OCT and OCTA as novel modalities

**Fig. 2:**
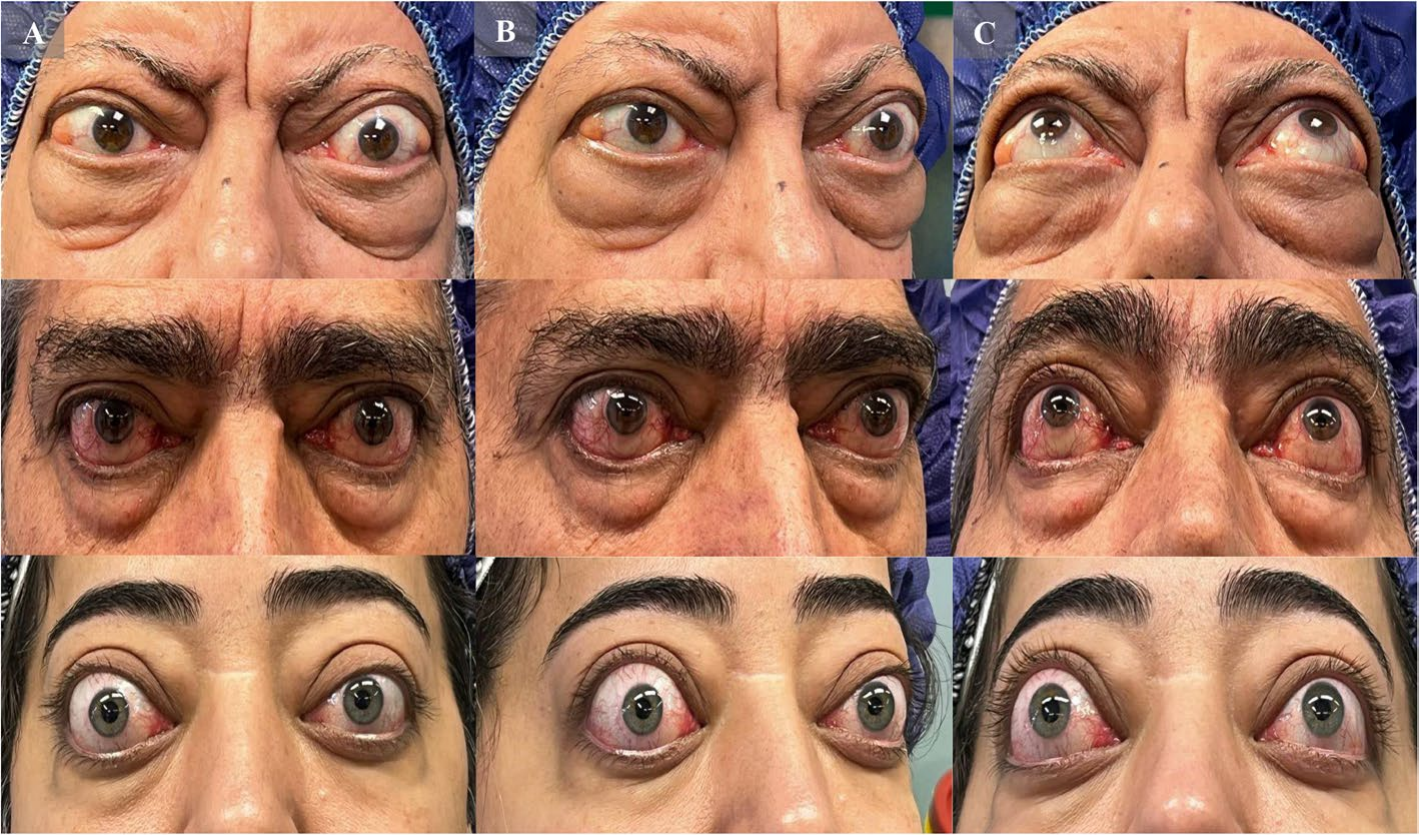
Clinical Photographs of three patients with active TAO in different views, Column A: Primary Position, Column B: Side view, Column C: Worm’s eye view. As seen in the pictures, patients present with severe proptosis, eyelid edema, and erythema with eyelid retraction

Early diagnosis of TAO is crucial to prevent further exacerbation of the disease and clinical complications. Three pillars of TAO diagnosis consist of clinical signs and symptoms (including eyelid retraction, proptosis, lid lag, eyelid edema, limited eye movements, in addition to exposure keratopathy and CON in severe cases) (Fig. 2), disruption of thyroid function tests (as TAO is accompanied by hyperthyroidism in 90% of cases) and imaging findings (such as application of CT, MRI and ultrasound findings especially in subjects with inconclusive clinical presentations) [25, 26].

Cross-sectional CT and MRI are important diagnostic tools in ophthalmologic disorders as they are first-line imaging tools for detecting orbital infections and soft tissue disorders respectively [27]. These imaging modalities have been applied in the detection of TAO; for instance, EOM enlargement, fat expansion, bony orbit remodeling, SOV enlargement, and anterior displacement of lacrimal glands are some of the critical features of TAO observed in CT scan imaging. MRI can also depict the same anatomical structures, and it is preferable for discovering soft tissue-related disorders and distinguishing active and stable phases of the disease [28]. Despite the significant role of these imaging modalities in initial diagnosis, surgical planning, and differentiation of TAO from other orbital disorders, there is not a clear consensus over which imaging method is best suitable for diagnosis of TAO [29].

However, CT is the standard modality for diagnosing and managing TAO[30]. Other imaging modalities are being applied in the setting of this disease, such as ultrasonography, which can be an important screening method for TAO by assessing EOM thickness [31].

Optical imaging modalities have been revolutionized in the past two decades, especially following the invention of optical coherence tomography (OCT) in 1991. OCT is a non-contact, non-invasive imaging modality of anatomical structures based on low coherence interferometry for measurement of reflected light, which can generate real-time cross-sectional images [32, 33]. OCT is an excellent modality for imaging the anterior eye and retina, which can also provide reliable intraoperative guidance during surgeries [34].

Despite the significant advancements of OCT, it could not provide useful functional imaging of moving anatomical structures upon a static background tissue, such as imaging small vascular structures with a proper contrast. Previously, intravenous dye-based imaging modalities such as FA and ICGA were mainly applied to provide imaging of retinal and choroidal microvasculatures, respectively. The issue with these methods was that both of them were time-consuming and both provided two-dimensional angiograms. OCTA as a significant branch of OCT, was a non-invasive dye-free imaging technique that provided the opportunity for performing three-dimensional en face (the best view for visualization of vascular tree) depth-resolved imaging of retinal and choroidal micro-vasculatures in addition to the ability for segmentation and distinguishing of anatomic layers using diffractive particle movement of red blood cells (RBC) [35, 36]. Furthermore, followed by the signal generation due to the movement of erythrocytes, the signal-to-noise ratio is improved via the split-spectrum amplitude-decorrelation angiography (SSADA) algorithm and splitting of source spectrum into four divisions. Central macular vasculature is a capillary bed consisting of three layers. Contrary to fluorescein angiography which cannot visualize deep and intermediate capillary plexuses explicitly, the application of OCTA provides an excellent opportunity to visualize these layers and the potential pathologic processes that can affect them such as ischemia. OCTA, in addition to providing detailed imaging of anatomic structures and microvasculatures, provides the opportunity for a comprehensive analysis of tissue perfusion, which is helpful for the detection of alterations before morphologic changes [37].

Unlike Doppler OCT, which was appropriate for imaging larger blood vessels and measuring their blood velocity using phase-shift, OCTA visualized vessels down to the capillary level. OCTA has proven effective for the detection of neovascularization, ischemia, capillary loss, absence or alterations of blood flow, irregular vascular geometry, and confirmed cases of diabetic retinopathy, age-related macular degeneration (AMD), and glaucoma [34, 38].

Quantitative analysis of microvascular features such as vessel density (VD), fractal dimension (FD), and foveal avascular zone (FAZ) was made feasible using OCTA; VD, FD, and FAZ being defined as the ratio of vessel occupied compared to total area, retinal microvasculature applicable geometrical index of complexity, and capillary network surrounded central area with no blood vessels respectively[39].

Based on previous studies, TAO is associated with an increase of choroidal vascularity index (CVI), mean and subfoveal choroidal thickness (SFCT); therefore, measurement of these indices using an imaging modality such as OCT could be useful for the detection of TAO. Moreover, the application of OCTA can be a significant tool for the assessment of the vascularity of the optic nerve and central retina in TAO patients[40, 41].

The OCTA technique applied for macular imaging used several transverse and axial scans, mapping a 6.0 mm × 6.0 mm area for macular scans. En face imaging contains a 3.0 mm × 3.0 mm fovea-centered section. The fovea and parafovea were identified as a 1.0 mm × 1.0 mm central avascular area and the annulus from 1.0 mm o 3.0 mm ring, respectively [38, 42].

### Changes of choroidal and retinal vasculatures in TAO

In our reviewed articles, participants first underwent a comprehensive ophthalmologic examination. TAO patients were categorized based on clinical activity score (CAS); most studies considered CAS equal to or greater than 3 as active disease. The severity of the disease was also assessed based on NOSPECS or EUGOGO classification. Additionally, cases with concomitant ocular disease or systemic diseases affecting the eyes, such as diabetes and hypertension, were excluded from the studies.

TAO, an autoimmune disorder, can affect ocular vascular structures via both inflammatory processes and increased intraocular pressure; therefore, several studies have evaluated changes in choroidal and retinal vasculature associated with TAO, as summarized in Supplement file, Table 3.

*Some studies focused on assessing changes in vessel densities in superficial and deep retinal layers:* foveal, parafoveal, and perifoveal. In some studies, all grid session evaluation was performed. Changes in the FAZ area were also evaluated in many studies. Compared to inactive TAO patients and healthy controls, significant enlargement of the FAZ area was reported during active TAO [43–45]. Although many studies reported no significant changes in FAZ area in inactive TAO patients compared to healthy controls [46–48], some studies claimed FAZ area to be increased in inactive TAO patients compared to healthy subjects [43, 49], in overall suggesting FAZ area to be significantly greater in TAO patients [45, 50].

*Some studies mainly focused on changes in perfusion density rather than vessel density.* TAO patients were reported to have increased macular and foveal PD (MPD and FPD) compared to controls, FPD being positively associated with CAS and MPD being inversely correlated with TAO severity [51]. Inactive TAO patients also had significantly higher mean and inferior PD measures of SRL [43]. SRCP PD and DRCP PD measures were significantly reduced in active and stable TAO patients compared to controls [50]. Peripapillary PD, ONH, and RPCP parameters significantly differed between TAO patients and controls. Additionally, DRCP-wPD, RPCP-wPD, SRCP-wPD, and ONH-wPD of OCTA-related parameters had the highest distinguishing ability for detecting active and control eyes [50]. Changes in PD between superficial and deep capillary plexuses (sCPD and dCPD) were also observed by initiating corticosteroid therapy for active TAO patients [52]. Pinhas et al.[53] also confirmed the remarkable reduction of non-capillary peripapillary PD in NDON TAO patients. (contrary to capillary peripapillary PD).

*Another important category of parameters in our reviewed articles was the factors associated with changes of the ONH area*.

In general, many studies reported a marked reduction of RPC density in TAO patients [44, 46, 50, 54], especially in the active phase of the disease [50, 55–57]. TAO patients with DON were reported to have a more significant decline in RPC density than the NDON patients compared to controls [54, 57, 58]. Furthermore, inactive (stable) TAO patients were reported to have lower RPC density compared to healthy subjects [46, 50]. Whole and superior RPC density were also remarkably reduced in patients with VF defect compared to patients without VF defect [59].

As of the other ONH-associated evaluations, a significant reduction of onh-wiVD and peripapillary VD in DON patients compared to NDON patients and healthy subjects was reported in many studies [58, 60–62]. Peripapillary vascularity index, peripapillary VD, ONH-wiVD, inside disc AV, and SV were also significantly declined in active TAO patients compared with stable patients and controls [55, 56, 63, 64].

As for ONH associated perfusion density changes, active patients had declined ONH average PD in the inferior, superior, and whole areas [65], and TAO patients with VF defect also had reduced retinal peripapillary PD [59].

*Some studies also evaluated the blood flow of choriocapillaris.* TAO patients had significantly decreased choriocapillary vascular flow [46]. Yilmaz et al. also confirmed the reduction of CC flow in the inactive TAO group compared to controls [49]. Mihailovic et al., however, found no significant changes in CC VD between inactive TAO patients and healthy subjects [46]. Erogul et al.[44] also found no significant changes in CC and outer retina flows between active TAO patients and controls. As for the CC changes followed by TAO treatment methods, CC BFI was significantly improved after two months of pulse therapy [66]. Foveal and superior-Hemi superficial CC VD was significantly reduced following decompressive surgery in NANC patients [67].

*Due to the potential probability of TAO leading into DON, many studies compared the changes in ocular vascular structures between DON TAO patients, non-DON patients, and healthy controls.*

6% of TAO patients develop DON, which is the most important complication of TAO due to vision impairment and loss in 30% of cases. However, early detection and treatment of DON in the reversible state is vital to retrieve visual function and prevent permanent optic nerve damage [5].

DON eyes had significantly deceased onh-wiVD and rpc-wiVD compared to NDON patients and healthy subjects, in addition to the reduction of macular wi-VD in all grid sessions compared to controls [60]. DON patients also had reduced DRCL density, RPC VD, peripapillary VD, and total and regional peripapillary RNFLT compared to NDON and control subjects.[54, 57, 58, 61, 62, 68]. GCCL thickness and SRCP density were also significantly reduced in DON patients compared to NDON and healthy subjects [65].

*The activity status of TAO was also an essential factor affecting ocular vasculature-associated changes in OCTA parameters.* Active TAO patients had significantly declined RNFLT, RPC density, peripapillary vascularity index, peripapillary ONH VD, mean temporal and inferior SLR VD, whole macular and parafoveal superficial VD, outer and inner macular vascularity index, DRCP PD, and increased FAZ area and Increased choroidal vascularization, retrobulbar blood flow, mean and central DVD (m-DVD and c-DVD) compared to inactive TAO patients [43, 45, 50, 55, 69]. However, active and inactive TAO patients observed no significant difference in peripapillary flow, RNFLT, SFCT, parafoveal SVD, and DVD parameters [45, 70]. Although, Sun et al. reported no changes in SVD and DVD between active and inactive patients [69]. Several studies reviewed the changes in the OCTA measures between inactive TAO patients and controls [43, 45–49, 57, 62, 63, 71]. A significant decrease of superficial ONH VD, RPC VD, macular superficial VD, and significant elevation of parafoveal VD was observed in inactive TAO patients compared to healthy subjects; however, there was not a significant difference in FAZ area, perimeter measurements, peripapillary VD, foveal, superior, inferior parafoveal and CC VDs, outer and inner macular vascularity index between groups [46, 47, 49, 63, 71]. Despite the reduction of SCP-VD measures in inactive TED patients compared to controls, there was no notable changes of DCP-VD and FAZ area in these patients.

Some studies, however, reported a significant increase of FAZ, mean, temporal and inferior VD, and PD of SRL in addition to a reduction of central macular thickness and VD of whole image, foveal, parafoveal and perifoveal regions in inactive patients compared to controls [43, 49]. Furthermore, No significant changes in ONH and RPC densities, FAZ area, acircularity index, macular SCP, and DCP were observed in inactive pediatric inactive TAO patients compared to controls [72].

Moreover, CAS values were positively correlated with FVD and FPD measures. TAO severity grade was also negatively correlated with foveal VD (FVD) and macular PD (MPD) [51]. TAO-associated VD decline was mainly correlated with disease activity more than the orbitopathy stage [67]. Lastly, there was no significant correlation between retinal VD and NOSPECS classification [61]. CAS values were also correlated with changes of RCD in TAO patients [73].

*Some studies also reviewed the correlation between changes of OCTA parameters and parameters associated with visual field such as BCVA, VF MD, etc*.

In our reviewed articles, the study population underwent ophthalmologic examinations to assess whether changes in ocular vasculature during TAO can lead to visual impairment. Some of the most critical evaluated parameters were BCVA, exophthalmometry (Hertel exophthalmometer), anterior and posterior segment slit-lamp examination, IOP, spherical equivalent (SE), axial length (AL), VF-MD, P100 amplitude, and latency.

BCVA was confirmed to be negatively correlated with RNFLT, TAO duration, whole image, and peripapillary VD [54, 71, 74]. P100 amplitude was positively correlated with macular and ONH whole image VD in addition to SLR and RPC whole VD; it was also negatively correlated with RNFLT [60, 74]. P100 latency was positively correlated with CRT [50].

VF-MD was negatively correlated with RNFLT, CRT, onh-wiVD, RPC density, macular and parafoveal whole superficial vasculature [50, 55, 59, 60, 62, 74]. IOP was correlated positively with the FAZ area and negatively with ONH-wiVD and RPC-VD [43, 58]. There was a significant correlation between IOP peripapillary VD and RNFLT [61]. AL was negatively correlated with FAZ [43]. Macular superficial microvascular densities and peripapillary capillary VD loss were associated with visual impairment [58, 75].

Reduced RCD measures among TAO patients compared to normal subjects, were associated with changes of visual acuity in addition to presence of increased IOP and proptosis in these patients [73].

The measurement of whole eye movements (WEM) was also an effective means of follow-up of inactive TAO patients, as there was a significant correlation between measures of WEM and whole peripapillary and SCP parafoveal VDs after adjustment by age, gender, AL and IOP, which indicates a significant association retinal VD decline and orbit stiffness in these patients [76].

*Some studies evaluated the potential involvement of smoking in the exacerbation of TAO through measurement of OCTA parameters*.

Smoker TAO patients had significantly reduced parafoveal, temporal, inferior hemi-parafoveal superficial VD, and total peripapillary VD compared to non-smokers. Passive smoker TAO patients had the highest RPC and ONH VD compared to active and non-smokers. However, superficial whole macular VD and deep retinal VDs were not significantly different between controls and active/passive smokers [48, 77]. Both smoker and non-smoker TAO patients had significantly higher FAZ acircularity index than healthy controls [48].

*As corticosteroids were confirmed as one of the essential pillars of TAO treatment, especially in the active phase of the disease, some studies evaluated the changes in OCTA parameters following corticosteroid therapy*.

Due to the importance of glucocorticoids in the treatment of TAO (especially in the active state of disease), some studies evaluated the changes of OCTA parameters following corticosteroid therapy [52, 62, 66]. Pulse therapy of active TAO patients remarkably improved BFI of deep macular plexus, external retina, and choriocapillaris and improved CAS and Hertel exophthalmometry measures, but superficial plexus BFI showed no significant changes [49, 66]. After treatment with IV methylprednisolone, TAO patients showed increased BCVA and decreased TSHR Ab, the superiorhemi-sector RNFLT, IOP, and proptosis but no significant changes in RPC density [62]. Furthermore, systemic IV corticosteroid therapy led to an increase of sFAZ, sCPD, dCPD, and reduction of dFAZ, in addition to improvement of chorioretinal blood flow, CAS, serum autoantibody levels and extraocular muscle thickness at the start of treatment [52]. Fan et al. also reported notable improvement of reduced macular VD and visual field of DON patients subsequent to application of high-dose IV MTP therapy [78].

*Following decompression surgery:* NDON and stable TAO patients who underwent surgical decompression, were confirmed to have significantly elevated Cup/Disc horizontal ratio, peripapillary SV density of RPC, and significantly reduced foveal superficial retinal VD, superficial CC VD postoperatively [67]. In an another study by Wu et al.[79] DON patients underwent IV corticosteroid therapy, and the ones not responding to steroid therapy underwent surgical decompression; as a result of these treatment approaches VF was significantly improved. However, there wasn't any immediate notable changes of choroidal RPC density following treatment.

Lastly, Zeng et al.[74] compared OCTA parameters between TAO patients with and without chorioretinal folds. CRF patients had significantly reduced RPC-wiVD, peripapillary wiVD, whole image, parafovea, perifovea, and all grid sessions VD of SLR; altogether, decreased retinal microvascular density in TAO patients with CRF could lead to visual impairment. Another study also confirmed that TAO patients with CRFs have significantly reduced vascular density of choriocapillaris, superficial and deep retinal layers compared to normal population. CRF patients with presence of edema in optic disc had also notably declined choriocapillaris density compared to non-ODE CRF patients [80].

### The applications of AI and future directions

Artificial intelligence (AI), through its wide range of applications in data analysis, has revolutionized several fields of medicine in recent years, its effects being more prominently reflected in radiology and pathology. The advancement of AI has been accompanied by the emergence of novel means of screening, diagnosis and treatment of the diseases, especially in the areas using imaging modalities as a main pillar of diagnosis and evaluation of the disease [81, 82].

The development of state-of-the-art AI-based technologies, such as machine learning (ML), deep learning (DL), support vector machines (SVMs), and convolutional neural networks (CNN), has influenced ophthalmology, similar to other fields of medicine. The technologies mentioned above have changed the course of data processing and perception of the information collected from imaging modalities in ophthalmology, including the data obtained from various imaging modalities such as fundus photography, ultrasonography, CT scan, MRI, OCT, and OCTA [81, 83].

TAO, being the most important autoimmune orbital disease, has been the focus of some AI-related studies, including the application of CNNs for diagnosis and assessment of TAO severity using orbital CT scan imaging, application of deep CNNs (DCNN) in differentiation of active and inactive TAO based on orbital MRI imaging or evaluation of DON among TAO patients, orbital CT scan based screening of TAO via 3D-ResNet technology and detection of EOM enlargement via ResNet-50 and VGG-16 technologies among TAO patients [84–89].

AI has also come to the aid of imaging modalities in the assessment of treatment outcomes of TAO patients, such as evaluation of the response to corticosteroid therapy via LRDTSVM-based interpretation of MRI images or the use of generative adversarial networks (GAN) for predicting the appearance of TAO patients prior to decompressive surgery [90, 91].

As mentioned before, TAO can affect orbital vascular structures, causing alterations of retinal and choroidal microcirculation; these changes can be studied comprehensively via measurement of quantitative OCTA parameters, including the parameters associated with the extent of FAZ, and evaluation of density, caliber, perimeter index and complexity index of blood vessels. These quantitative OCTA measures can decrease subjective human error concerning screening of highly detailed retinal images in addition to being more sensitive for detecting subtle microvascular alterations in the early stages of the diseases. It could be inferred that, by applying an appropriate means of data analysis, OCTA measurements contain substantial information that can be applied to detect retinopathies and differentiation of disease stages. By utilizing AI-based digital image processing and learning technologies, the data obtained from OCTA can be classified and analyzed systematically and organized, such as the novel applications of CNNs for early detection of retinopathies by means of OCTA images [92, 93].

Another OCTA-related feature that is highly sensitive for staging retinopathies is the artery-vein (AV) analysis. AV classification, while necessary for disease diagnosis, lacks the feasibility for clinical application due to the high complexity and large sizes of imaging data. However, the application of DL algorithms can facilitate the automation of AV classification. DL has also proven effective for automatically segmenting areas of interest in OCTA, such as avascular regions, including FAZ [94, 95].

To summarize the possible applications of AI in combination with OCTA, it can be stated that AI, especially learning algorithms associated with it, can be helpful in OCTA signal generation, reduction or removal of artifacts, enhancement, and reconstruction of OCTA images, segmentation of retinal slabs, comprehensive analysis of OCTA associated features, and analysis vascular and perfusion densities [96–98].

Learning algorithms have been applied to create OCTA-like angiograms from OCT scans and differentiate flow and static voxels with a smaller image acquisition duration. Some important studies indicate the use of CNNs to increase the image quality of high-definition OCTA scans. Many DL-based algorithms have also been successfully applied in retinal slab segmentations, with high accuracy for separately analyzing important areas such as the macula, ONH, and choroid [98].

Altogether, the novel advancements of AI, especially in deep learning algorithms, can potentiate OCTA imaging to be further involved in the screening, diagnosis, and assessment of treatment outcomes in ophthalmologic diseases affecting the retinal and choroidal circulation. However, at present, we could not find any studies regarding the application of AI-based algorithms in the analysis of OCTA images of TAO patients. We speculate that applying the AI and DL-based algorithms for evaluation and analysis of the data obtained from OCTA images of TAO patients can promise remarkable opportunities in regards to novel algorithms of early detection and diagnosis, assessment of activity and severity, prevention of DON, and evaluation of response to treatments in TAO patients.

## Conclusion

**Fig. 3:**
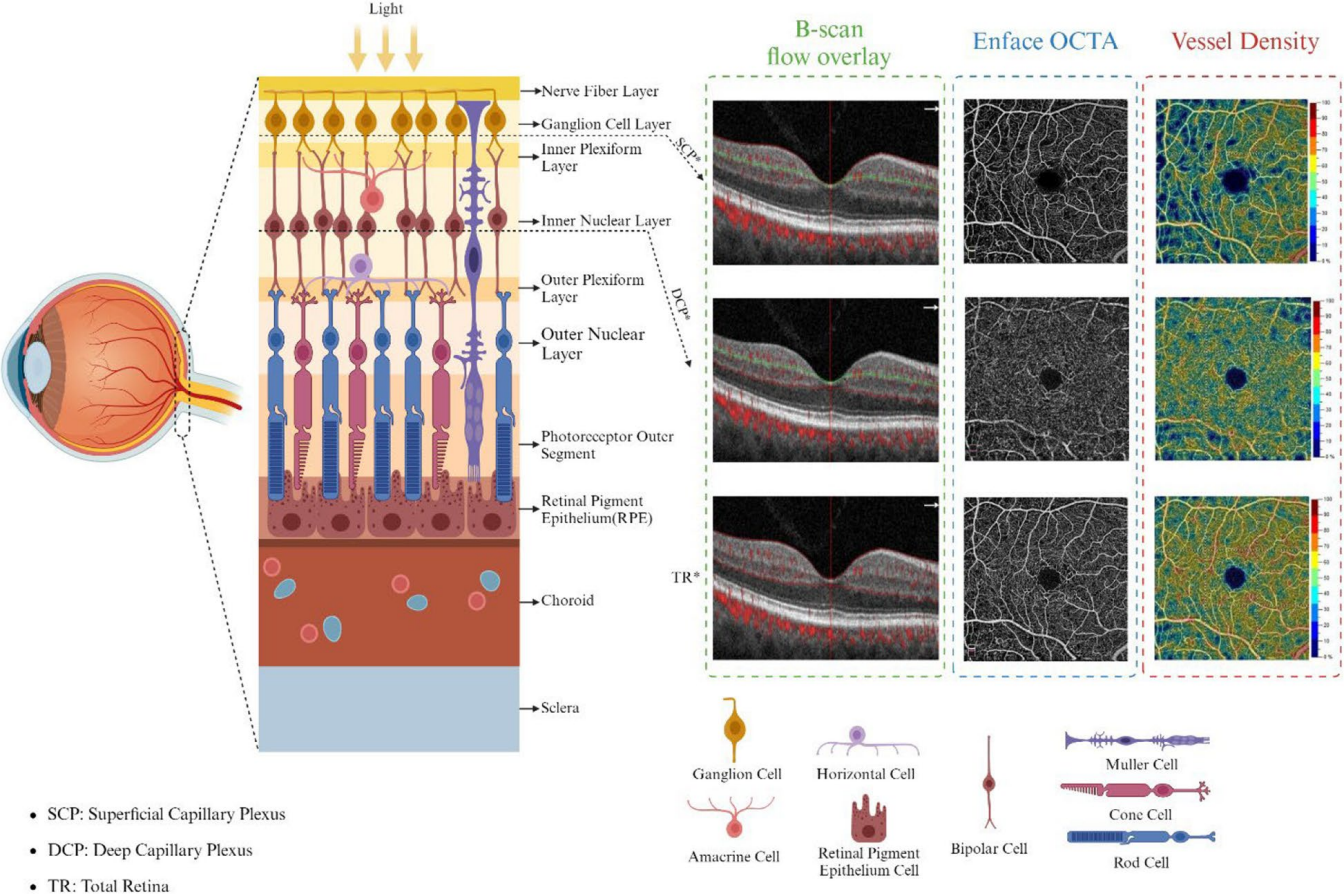
A comparison between OCTA images in different retinal layers (Created with BioRender.com)

**Fig. 4:**
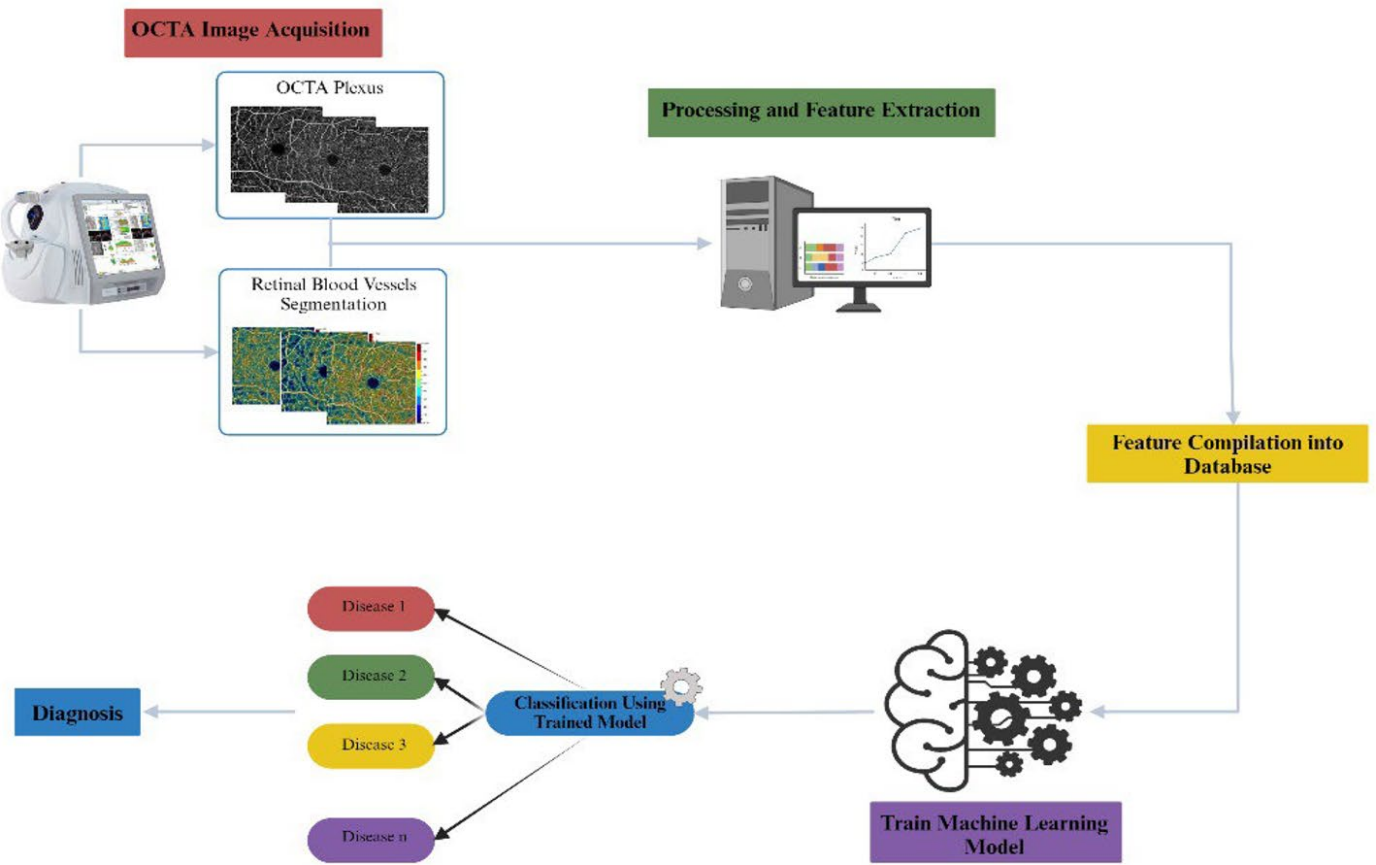
a summary of the basic steps in machine learning. After image acquisition, features are extracted, digital images are processed and the data are compiled in a database. Then, the dataset is used to train the machine learning model to carry out the classification task for either distinct diseases or staging. (Created with BioRender.com)

TAO, the most common immune orbital disorder, can affect ocular hemodynamics and vascular structures of the eye, including choroidal, retinal, and optic nerve surrounding microvasculature. In this study, we reviewed all the studies that evaluated the alterations of ocular vasculature-associated parameters using OCTA. OCTA measurements provide valuable information regarding changes of vessel density and perfusion density of macular, foveal, parafoveal, and peripapillary regions in addition to the difference of FAZ area, RPC density, choriocapillaris blood flow, etc., through comparison of TAO patients and healthy subjects. Furthermore, in some studies, these parameters are also evaluated in association with disease activity, presence of DON, smoking, corticosteroid therapy, etc (Figs. 3 and 4).

### Supplementary Information

Supplementary file 1.

## Data Availability

The datasets used in the current study are available upon reasonable request.

## Abbreviations

TED: Thyroid eye disease
TAO: Thyroid-associated ophthalmopathy
GO: Graves’ orbitopathy
EOM: Extraocular muscles
ON: Optic nerve
ONH: Optic nerve head
SOV: Superior ophthalmic vein
IOP: Intraocular pressure
DON: Dysthyroid optic neuropathy
TSHR: Thyroid stimulating hormone receptor
IGF-1R: Insulin-like growth factor-1 receptor
RBP: Retrobulbar pressure
CAS: Clinical activity score
EUGOGO: European Group on Graves’ Orbitopathy
CON: Compressive optic neuropathy
FA: Fluorescein angiography
ICGA: Indocyanine green angiography
SSADA: Split-spectrum amplitude decorrelation angiography
VD: Vessel density
FD: Fractal dimension
FAZ: Foveal avascular zone
FAZ AI: FAZ acircularity index
CVI: Choroidal vascularity index
CT: Choroidal thickness
SFCT: Subfoveal choroidal thickness
SRL: Superficial retinal layer
DRL: Deeper retinal layer
MIR: Retinal microvascular
STMI: Superficial total MIR
SMIR: Superficial MIR
DMIR: Deeper MIR
RPC: Radial peripapillary capillary
rpc-wiVD: Radial peripapillary capillary whole image vessel density
onh-wiVD: Optic nerve head whole image vessel density
m-wiVD: Macular whole image vessel density
VF-MD: Visual field mean defect
BCVA: Best-corrected visual acuity
AL: Axial length
NANC: Not active not compressive
GCC: Ganglion cell complex
GCCL: Ganglion cell complex layer
GCCT: GCC thickness,
CC: Choriocapilaris
RNFLT: Retinal nerve fiber layer thickness
NFL: Nerve fiber layer
SRCL: Superficial retinal capillary layers
DRCL: Deep retinal capillary layers
RCD: Retinal capillary density
PD: Perfusion density
BFI: Blood flow index
DP: Deep plexus
ER: External retina
PVBFI: Peripapillary vascular blood flow indices
PP: Pulse pressure
AV: All vessels
SV: Small vessels
MTP: Methylprednisolone
SVD and DVD: Superficial and deep VDs
TRAB: Thyroid stimulating hormone-receptor autoantibodies
PPCMv: Parapapillary choroidal microvasculature
CVI: Choroidal vascularity index
