# Supplementary material for: Optical coherence tomography angiography in thyroid associated ophthalmopathy: a systematic review
Source: BMC Ophthalmol. 2024 Jul 22;24:304. doi: 10.1186/s12886-024-03569-5 (PMC11265183; doi:10.1186/s12886-024-03569-5)
Supplement: Supplementary file 1 [file 12886_2024_3569_MOESM1_ESM.docx]

**Table. 1** Clinical Activity Score Components for Assessment of TAO

| CAS-based TAO activity assessment | |
| --- | --- |
| 1 | Spontaneous retrobulbar pain |
| 2 | Pain on attempted upward or downward gaze |
| 3 | Redness of eyelids |
| 4 | Redness of conjunctiva |
| 5 | Swelling of caruncle or plica |
| 6 | Swelling of eyelids |
| 7 | Swelling of conjunctiva (chemosis) |

**Table. 2** EUGOGO Classification for Assessment of TAO Severity

| EUGOGO Classification | Characteristics |
| --- | --- |
| Mild | Patients with little influence of TAO on daily life without justification of surgery or immunotherapy with one or more of the following:  • Lid retraction (<2 mm)  • Mild involvement of soft tissue  • Exophthalmos<3 mm above normal for race and gender  • No or intermittent diplopia |
| Moderate to Severe | TAO patients with a greater burden of disease in daily life who have the indication of surgery (if inactive) or immunosuppression (if active); with two or more of the following:  • Lid retraction ≥ 2 mm  • Moderate or severe involvement of soft tissue  • Exophthalmos ≥ 3 mm above normal for race and gender  • Inconstant or constant diplopia |
| Very Severe (sight-threatening) | TAO patients with DON or corneal breakage |

**Table. 3** Review of Literature for the changes of OCTA parameters in TAO

| **No.** | **Study** | **Subjects** | **OCTA** | **Assessment of** **radial peripapillary capillary (RPC) density** | **Assessment of other optic Nerve associated OCTA parameters** | **OCTA Parameters associated with choroid and macula** | **Important Correlations Found** | **Summery and conclusion** |
| --- | --- | --- | --- | --- | --- | --- | --- | --- |
| 1 | Ye et al.(43)  2018 | 20 active TAO patients  20 normal controls (Right eyes) | Three-dimensional OCTA images (RTVue Avanti XR system (Optovue, Fremont, CA) with 6 × 6-mm angiographic OCT scans of the superficial retinal layer (SRL) and deeper retinal layer (DRL) of the macula | Not assessed | Not assessed | ⏵Increased measures of retinal microvascular (MIR) density in active TAO patients, in addition to the increase of STMI (superficial total MIR) and SMIR (superficial MIR) density  ⏵Significantly increased DMIR (deeper MIR) of the deep layer in active TAO  ⏵No significant changes in MAR (macrovascular) density  ⏵No notable quadrant changes of SMIR and DMIR | Potential correlation of macular STMI and SMIR with disruption of visual acuity | Elevated SMIR and STMI of TAO patients with increased retinal MIR density can affect visual acuity. |
| 2 | Lewis et al.(44)  2018 | 12 moderate to severe GO patients (24 orbits) undergoing bony orbital decompression surgery, 4 of the patients (6 orbits) with DON | Angio Vue OCT-A, software on the Avanti RTVue-XR spectral-domain  OCT system (Optovue, Inc; Fremont, CA), a 4.5 × 4.5 mm ellipsoid scan surrounding the ON | Not assessed | ⏵Reduced VD of optic disc and peripapillary areas in most of the DON eyes (this proportion was lower in NDON eyes)  ⏵Lowered inside disk area mean VD in most eyes postoperatively (but only about half of them exhibited this reduction in the peripapillary ring postoperatively)  ⏵Higher values of mean CAS and exophthalmometry measurements in the DON group compared to NDON patients  ⏵Clinically improved DON eyes following surgery had a much higher decline of the VD of the area overlying the optic disk compared to NDON eyes | Not assessed | No other significant correlations were found | Optic nerve-related VD alterations of GO patients could indicate vascular diameter changes after retrobulbar orbital pressure. Furthermore, OCTA can be effective in monitoring the progression of GO and the risk of DON, in addition to analyzing the outcomes of medical and surgical treatments. |
| 3 | Zhang et al.(45)  2019 | 41 TAO eyes without DON, and 30 with DON  23 healthy eyes | A single OCT-A system (AngioVue; Optovue, Inc., Fremont, CA, USA) with a 4.5×4.5-mm cube angio scan for evaluation of the optic nerve head (ONH) and a 3.0×3.0-mm macular cube angio scan for evaluation of the fovea | Significantly reduced radial peripapillary capillary whole image vessel density (rpc-wiVD) in DON eyes compared with healthy and non-DON eyes | Significantly reduced optic nerve head whole image vessel density (onh-wiVD) in DON eyes compared with healthy and non-DON eyes | ⏵Significantly decreased macular whole image vessel density (m-wiVD) of NDON patients compared to controls in all grid sessions other than the fovea  ⏵No remarkable difference in macular VD parameters between DON and NDON eyes | ⏵ Positive correlation of m-wiVD and P100 amplitude  ⏵Positive correlation of onh-wiVD and rpc-wiVD with visual field mean defect (VF-MD) and P100 amplitude | A significant decrease was observed in the measures of radial peripapillary capillary whole image vessel density (rpc-wiVD) and optic nerve head whole image vessel density (onh-wiVD) in DON eyes compared with healthy and non-DON eyes with a more significant decline in the temporal peripapillary. There was a positive correlation between visual function deterioration and the reduction of onh-wiVD and rpc-wiVD. The scope of visual impairment was remarkably associated with attenuation of peripapillary perfusion. |
| 4 | Jamshidian_Tehrani et al.(46)  2019 | 12 active TAO patients (21 eyes)  43 not active not compressive (NANC) patients (77 eyes)  35 healthy controls (65 eyes)  12 active TAO patients (21 eyes)  43 NANC patients (77 eyes)  35 healthy controls (65 eyes)  (NANC defined as CAS≤3 and active TAO as CAS>3) | The RTVue Avanti spectral-domain OCT (AngioVue, Optovue, Inc., Fremont, CA) for the evaluation of the peripapillary and macular areas via a 6 × 6 mm macular scan for measurement of superficial and deep macular capillary plexuses, and a 4.5 × 4.5 mm rectangle scan centered on the optic disc for the evaluation of optic nerve | Significantly reduced whole image, total, nasal, temporal, and infratemporal RPC density in the active group compared to NANC and controls | Significantly reduced whole image, total, and nasal peripapillary ONH VD in the active group compared to NANC and controls | ⏵Significant reduction of whole macular and parafoveal superficial vessel densities in the active TAO  ⏵Lowered parafoveal vasculature value in NANC TAO group in the nasal sector only  ⏵Reduced deep layer vessel densities of the active group compared to controls | Association of VF-MD with both macular and parafoveal whole superficial vasculature, but not with the deep macular and parafoveal  vasculature. | Despite no significant differences in macular ganglion cell complex thickness (GCCT) among the study groups, subclinical retinal and optic nerve involvement was confirmed in the active TAO eyes based on decreased peripapillary and macular vessel density values. |
| 5 | Mihailovic et al.(47)  2019 | 29 inactive GO patients (29 eyes)  29 healthy controls (29 eyes) | RTVue XR Avanti with AngioVue (Optovue, Inc, Fremont, California, USA) using a 4.5 ×4.5 mm2 scan of ONH and a 3 × 3 mm2 scan of macula | Significantly reduced RPC density in GO | Significantly reduced ONH superficial VD in GO | ⏵Significantly reduced macular superficial VD in GO (whole en face and parafoveal density)⏵No significant changes in the FAZ area  ⏵No significant changes of choriocapillaris (CC) VD | No significant correlations of VD and NOSPECS classification, MD, and PSD of Humphrey visual field testing | Changes in ocular perfusion, including markedly reduced RPC, macular, and ONH superficial VD were observed in GO patients, unlike controls; however, there was no significant difference in FAZ area and CC VD between the two groups. |
| 6 | Wu et al.(48)  2020 | 44 TAO patients consisting of 23 NDON (36 eyes) and 21 DON patients (38 eyes)  38 healthy controls (38 eyes) | A commercial SD-OCT (spectral domain OCT) device (Optovue RTVue XR Avanti; Optovue, Inc.Fremont, CA, USA), OCT-A mode (3 × 3 mm area) for imaging of superficial and deep retinal capillary layers (SRCL and DRCL) of macular microvasculature | Not assessed | Not assessed | ⏵Reduced retinal capillary density (RCD) in the SRCL and DRCL of both TAO groups in comparison with the control group  ⏵Further lowered RCD in DRCL in DON patients compared to the non-DON group | No other significant correlations were found | Simultaneous significant decline in the thicknesses of the nerve fiber layer (NFL), ganglion cell layer, inner plexiform layer, and GCC was observed, as well as reduced RCD in the SRCL and DRCL of both TAO groups compared with the control group.  Both DON and NDON TAO patients had thinner intra-retinal layers and reduced microvascular density compared to controls. Morphologic changes such as in the NDON TAO patients suggest precedence of these changes to visual impairment. |
| 7 | Akpolat et al.(49)  2020 | 29 patients with inactive GO with CAS<3 (58 eyes)  30 age-matched healthy controls (60 eyes) | Triton model OCT-A (Topcon DRI OCT Triton swept-source OCT; Topcon, Tokyo, Japan), 3 × 3mm foveal scan | Not assessed | Not assessed | ⏵Significantly higher temporal and nasal parafoveal VDs in GO  ⏵No significant difference in foveal, superior, and inferior parafoveal VDs between groups | Strong negative correlation between GO duration and BCVA | Elevated values regarding IOP, proptosis, and axial length and significantly higher temporal and nasal parafoveal VDs, narrower retinal vein, and artery with a decreased artery/vein ratio were observed in GO patients compared to controls. There wasn't a significant difference in foveal, superior, and inferior parafoveal VDs between the two study groups. |
| 8 | Fazil et al.(50)  2020 | 36 healthy controls (36 eyes)  36 patients with inactive GO (36 eyes) | Zeiss Angioplex™ optical coherence tomography angiography (Carl Zeiss Meditec AG, Jena, Germany), a 6 × 6-mm macular scan area | Not assessed | Not assessed | No significant difference in VDs of SRL and DRL, FAZ area | No other significant correlations were found | Although there was an insignificant increase of CT in GO patients, VDs of SRL and DRL, FAZ area and perimeter measurements showed no significant difference between the two groups |
| 9 | Del Noce et al.(51)  2020 | 18 TAO patients, 10 active TAO and 8 inactive TAO patients (36 eyes)  18 aged matched patients (36 eyes) | OCT swept-source angiography scans were performed for evaluation of macular region CT via a 4.5 × 4.5 mm2 fovea-centered area | Not assessed | Not assessed | ⏵Significantly decreased CC vascular flow of TAO patients  ⏵Higher deeper choroidal layer flow in the TAO group | A significant correlation between the choroidal thickness and EUGOGO clinical score | Compared to control eyes, a significant increase in subfoveal choroidal thickness (CT) was observed in TAO patients. Furthermore, choriocapillary vascular flow significantly declined in the TAO group; however, the vascular flow in the deeper choroidal layer of TAO patients was higher than healthy subjects |
| 10 | Yu et al.(52)  2020 | 20 active TAO patients  33 inactive TAO patients  29 healthy controls | OCTA images obtained via Cirrus high-definition OCT prototype with AngioPlex (Carl Zeiss Meditec, Dublin, CA, USA) with a 3 × 3mm macular scanning pattern | Not assessed | Not assessed | ⏵Significantly enlarged FAZ area in active TAO patients compared to inactive and control groups, and in inactive patients compared to controls  ⏵Significantly larger mean, temporal, and inferior VD of SRL in inactive TAO compared to active patients and controls  ⏵Significantly higher mean and inferior PD of SRL in inactive TAO than the control group | ⏵Positive correlation of FAZ area with IOP  ⏵Negative correlation of FAZ area with axial length | The active TAO group had reduced retinal nerve fiber layer thickness (RNFLT) in addition to markedly increased mean FAZ area in OCTA-based assessment of superficial retinal vessels compared to other groups. A significant macular CT increase was observed in inactive and active TAO patients. Furthermore, inactive patients had significantly greater SRL vascular density compared to other study groups. The extent of the FAZ area and vascular and perfusion density (PD) were proven useful as diagnostic measures for distinguishing TAO/inactive TAO and inactive TAO/normal subgroups. |
| 11 | Dave et al.(53)  2020 | 24 eyes active TAO  102 eyes inactive TAO  52 healthy eyes | swept-source OCT system (Swept Source DRI OCT TritonTM, Topcon Medical Systems, Inc) via a 6×6 mm area fovea-centered scanning protocol and an ONH-centered 6 × 6 mm area | Not assessed | ⏵Significantly decreased peripapillary vascularity index in active compared to inactive TAO  ⏵Significantly higher peripapillary VD in active patients compared to inactive patients | ⏵Significantly reduced outer and inner macular vascularity index in active TAO compared to inactive TAO and controls  ⏵No significant difference in outer and inner macular vascularity index between inactive TAO and controls | Positive correlation of macular vascularity index with increased proptosis | In the active TAO group, peripapillary and the macular vascularity index declined compared to the other two groups, in addition to an increase of RNFLT in TAO eyes compared to inactive TAO and healthy subjects. |
| 12 | Jian et al.(54)  2021 | 43 TAO patients (4 unilateral and 39 bilateral, 82 eyes in total) were classified into active DON (ADON) or active non-DON (ANDON) and inactive NDON (INDON) groups according to CAS criteria.  26 healthy subjects (52 pairs of eyes) | Optovue Angio-VueTM (RTVue XR Avanti, Optovue Inc. Fremont,  CA. USA) a 4.5 × 4.5 mm scan area around ON | Not assessed | Significantly declined peripapillary VD among DON patients compared to other groups | Not assessed | ⏵Significant correlation of IOP, gender, thyroid condition, and smoking history to peripapillary VD and RNFLT  ⏵No significant correlation between retinal VD and NOSPECS classification | The total peripapillary RNFLT and regional peripapillary RNFLT (including the superior, inferior, and nasal sector peripapillary RNFLT) were markedly declined in the DON group. (no notable difference in these measures among other groups). Compared to other study groups, a significant increase of IOP in addition to a notable decline of peripapillary VD was also observed among DON patients; these results suggest OCTA-based assessment of peripapillary VD as a potential prognostic criteria of TAO-associated optic neuropathy in addition to confirming increased IOP as a risk factor of TAO related decreased retinal vessel density and NFLT. |
| 13 | Jamshidian-Tehrani et al.(55)  2021 | TAO patients were classified into 22 eyes of active smokers, 11 eyes of passive smokers, and 69 eyes of non-smokers (21 eyes of TAO patients had active TAO with CAS≥3, 77 were NANC eyes with CAS<3, and 4 eyes were categorized as compressive group). | RTVue XR Avanti SD-OCT system (Optovue, Fremont, CA, USA), a 6 × 6-mm macular centered and a 4.5 × 4.5-mm optic disc centered scan | Highest RPC VD among passive smokers (significantly in whole image RPC) | Highest ONH VD among passive smokers | ⏵Significantly lower parafoveal, temporal, and inferior hemi-parafoveal superficial VD in smokers compared to non-smokers  ⏵No significant changes of superficial whole macular VD and superficial VD of other parafoveal sectors among groups.  ⏵Significantly higher deep macular VD of non-smokers than the other groups  Significantly higher deep parafoveal VD of passive smokers compared to non-smokers  ⏵No significant changes of deep whole macular VD and deep VD of other parafoveal sectors among groups | No other significant correlations were found | Markedly elevated inferior hemi-parafoveal sector thickness of GCC, increased retinal thickness (except for the foveal area), and reduced superior parafoveal sector superficial VD were observed in smoker patients compared to non-smokers with no remarkable difference in deep VDs. Passive smokers had the highest RPC, macular and ONH VDs, with a significant difference in the whole image, infranasal peripapillary, and parafoveal sectors. |
| 14 | Ceylanoglu et al.(56)  2021 | 34 healthy controls  41 inactive GO patients (all with CAS<3, 22 smoker patients, and 19 non-smoker patients) | XR Avanti AngioVue OCTA (Optovue, Fremont, California, USA) (Version 2017.1.0.151) via a 4.5 × 4.5 mm rectangular optic disc centered and a 6 × 6 mm macular scan | Reduced RPC VD in superior and inferior sectors in smoker patients | Reduced total peripapillary VD in smoker patients | ⏵Significant increase of FAZ acircularity index (FAZ AI) in smoker and non-smoker GO patients  ⏵No significant difference in DCP, SCP, and FAZ area among groups | No other significant correlations were found | In smoker patients, RPC VD in superior and inferior sectors and total peripapillary VD were reduced compared to controls. A significant increase of FAZ AI was also observed in smoker and non-smoker GO patients compared to controls, all suggesting the effect of smoking on ONH microvasculature in inactive GO patients. |
| 15 | Del Noce et al.(57)  2021 | 24 patients (right eyes) who underwent pulse therapy with methylprednisolone (MTP). | Angio-OCT analysis (OCT Topcon ImageNet 6; DRI OCT Triton, Topcon Corporation), a 4.5 × 4.5 mm2 fovea centered scan | Not assessed | Not assessed | ⏵Significant improvement in macular blood flow index (BFI) of DP (deep plexus), ER (external retina), and BFI of CC following treatment  ⏵No significant improvement of superficial plexus BFI subsequent to treatment | No significant correlation of macular BFI (at any level) with CAS or visual acuity | After two months of pulse therapy, as well as improvement of CAS, macular BFI of DP, ER, and choriocapillaris were significantly improved. |
| 16 | Abdolalizadeh et al.(58)  2021 | 20 healthy controls (39 eyes)  13 Graves's patients without TAO (26 eyes)  14 patients with mild TAO (28 eyes)  17 patients with moderate-severe TAO (30 eyes)  12 patients with DON TAO (21 eyes) | AngioVue system (Optovue RTVue-XR 100 Avanti; Optovue, Inc., Fremont, CA, USA, software version 2017.1.0.151), A 4.5 × 4.5 mm rectangle optic disc centered scan | Declining trend of RPC VD in TAO patients (significantly moderate-severe TAO and DON patients) | Insignificant elevation of peripapillary and whole image VD from controls to Graves's patients without TAO | Not assessed | A significant correlation of worse BCVA, color vision, and VF with lower whole image and peripapillary VD | There was an unremarkable elevation of peripapillary and whole image VD from controls to Graves's patients without TAO; in TAO patients, RPC VD had a declining trend, which was significant in moderate-severe TAO and DON patients. Visual impairment, thinner GCC, and RNFLT were significantly associated with reduced peripapillary and whole-image VD. |
| 17 | Zhu et al.(59)  2022 | 36 active TAO patients  39 healthy controls | OCTA measurements for evaluation of ONH vessel density and pRNFLT using SD-OCT (RTVue XR Avanti, Optovue Inc. Fremont, CA. USA) and whole image and peripapillary fields scanning of ONH | Not assessed | Significantly reduced ONH-wiVD and peripapillary VD in active TAO patients compared to controls | Not assessed | Significantly increased IOP and lower BCVA in active TAO patients compared to controls | Among TAO patients, the fractional low-frequency fluctuation amplitude (fALFF) values of the left posterior cingulate gyrus (L.PCC) were correlated positively with peripapillary VD, these values were negatively correlated with the right calcarine (R.CAL) peripapillary VD, indicating that peripapillary microvascular changes in active TAO could elicit alterations in spontaneous brain activity associated with visual functions; however, there wasn't a significant correlation between changes in fALFF values and ONH-wiVD or pRNFLT. |
| 18 | Del Noce et al.(60)  2022 | 26 TAO patients (18 active and 8 inactive patients), 26 healthy subjects | OCT swept-source angiography scans obtained using OCT Topcon ImageNet 6 (DRI OCT Triton, Topcon Corporation) from a 4.5 × 4.5 mm2 ON centered area | Not assessed | ⏵Significantly decreased both DCP peripapillary vascular blood flow indices and (DCP-PVBFI) and choriocapillaris PVBFI (CC-PVBFI) in TAO patients compared to controls  ⏵No remarkable differences of SCP-PVBFI and outer retina PVBFI (OR-PVBFI) between groups  ⏵No significant differences between men and women in PVBFI values  ⏵No significant changes of macular morphology, RNFLT, and GCCT between patients and controls | Not assessed | Significant correlation of higher CC-PVBFI with CAS measures and TAO activity | TAO can affect indices associated with choriocapillaris and deep capillary plexus. Furthermore, the choriocapillary vascularity index was elevated in active patients compared to inactive ones, indicating the importance of peripapillary microvascular changes in DON development. |
| 19 | Pinhas et al.(61)  2022 | 8 NDON TAO patients with CAS of ≤ 3  (results compared to healthy controls of a previous study) | A commercial spectral-domain OCT-A system (Avanti RTVue-XR, Optovue, Fremont, California) for obtaining 4.5×4.5mm en face peripapillary scans | Not assessed | ⏵Significantly reduced non-capillary PD in the patients  ⏵No significant differences in capillary peripapillary PD | Not assessed | No significant correlations of disease-associated measures with OCTA PDs | Pathophysiologic pathways leading into TAO-associated vasculopathy can be involved in reducing blood flow and subsequent risk of ON ischemia. |
| 20 | Wu et al.(62)  2022 | 19 TAO patients with DON  24 non-DON TAO patients (NDON)  20 equivocal DON patients  34 healthy controls | RTVue XR Avanti spectral domain OCT system (Optovue, Inc., Fremont, CA, USA), a 4.5 × 4.5 mm ONH-centered scan | Significantly reduced RPC-VD in all areas of EDON patients | Significantly lower ONH-wiVD in EDON patients | Not assessed | ⏵Negative correlation of ONH-wiVD and RPC-VD with IOP  ⏵Correlation of peripapillary capillary VD loss with visual impairment | Despite no notable differences among the study groups in p-RNFLT, compared to healthy subjects, EDON patients had markedly reduced RPC-VD in all areas in addition to lower ONH-wiVD with more probability of temporal and upper regions VD for being affected by early TAO. The loss of peripapillary capillary VD was tightly correlated with visual impairment, as ONH VD (involving RPC-VD and ONH-wiVD) was affected by TAO. |
| 21 | Ye et al.(63)  2022 | 62 TAO eyes (39 with visual field (VF) defect and 23 without VF defect) | AngioVue; Optovue, Fremont, CA, USA OCTA system, ONH centered RPC imaging with a 4.5 × 4.5 mm scan size and fovea centered 3.0 × 3.0 mm scan for imaging of superficial retinal capillary plexus (SRCP), a 4.5 × 4.5 mm ONH centered scan | Significantly reduced whole and superior RPC density in patients with VF defect | Reduced retinal peripapillary vessel perfusion in patients with VF defect | No significant changes in whole, superior, nasal, temporal, or inferior SRCP density between the two groups | ⏵Correlation of RPC density with VF MD  ⏵Significant correlation between RPC density and pRNFLT | Among TAO patients, significantly decreased RPC density, elevated pulse pressure (PP), and vertical and horizontal muscle index were observed in the patients with VF defect compared to the other group. In TAO patients, decreased perfusion of retinal peripapillary vessels was associated with increased PP, suggesting the importance of systemic vascular alterations causing impairment of VF. In addition, this reduction of peripapillary vessel density was correlated with advancement of VF defect. SRCP density and pRNFLT were not remarkably different between the two study groups. |
| 22 | Yilmaz et al.(64)  2022 | 65 controls (65 eyes)  65 inactive TAO patients (65 eyes) | AngioVue Avanti RTVue-XR, OptoVue, Fremont, CA, using a scan size of 6 × 6-mm for macular imaging and a 4.5 × 4.5-mm scan for optic disc (OD) | Not assessed | No significant changes in peripapillary VD (other than the inferior sector of the peripapillary area) | ⏵ Significantly reduced central macular thickness and VD of whole image, foveal, parafoveal, perifoveal, superior, temporal, nasal, inferior, superior, and inferior Hemi SCP. (Same alteration pattern in DCP VD except for foveal DCP)  ⏵Reduced CC flow in TAO group  ⏵Increased FAZ in TAO patients | No other significant correlations were found | In addition to significantly elevated IOP and Hertel exophthalmometry measures in TAO group, significant macular VD decrease in all superficial foveal quadrants, superficial and deep parafoveal and perifoveal regions in addition to decrease of choriocapillaris (CC) flow and increase of FAZ was observed in TAO patients compared to controls; despite this reduction of parafoveal and perifoveal SCP and DCP, there wasn't a notable difference between two study groups in OD, whole image and peripapillary VD except for inferior sector of peripapillary area. |
| 23 | Sabermoghaddam et al.(65)  2022 | 28 healthy controls (28 eyes)  29 TAO patients (29 eyes) divided into group A: CAS<3 patients (18 patients) and group B: patients with CAS ≥ 3 (11 patients) | SD-OCT and OCTA images (AngioVue) were obtained via Optovue RTVue XR Avanti technology, ONH-OCTA for assessment. All vessels (AV) and small vessels (SV) VD inside disc and RPC, RPC imaging performed using AngioDisc 4.5 × 4.5 mm HD scan | Non-significantly declined RPC density in TAO patients (greater in patients with higher CAS score) | Significantly reduced mean whole image AV VD and SV VD in CAS B group compared to CAS A | Not assessed | Correlation of TAO activity with reduced VD of ONH or retina | CAS B group had a significant reduction of mean whole image AV VD and SV VD compared to CAS A group; however, there wasn't a remarkable difference between TAO patients and controls in mean whole image AV VD and SV VD. Regarding the changes in ONH microvasculature, TAO patients had a non-significant decline in RPC density, which was more profound in patients with higher CAS scores. |
| 24 | Erogul et al.(66)  2022 | 46 active TAO patients with CAS ≥3  41 healthy controls | The Optovue AngioVueTM, a 4.5×4.5 mm2 area for optic disc quantification | Significantly increased RPC density in temporal and inferior sectors in TAO patients | Not assessed | ⏵Significantly increased FAZ in TAO patients  ⏵Significantly reduced superficial foveal, parafoveal, and perifoveal VD in TAO patients  ⏵ Significantly reduced deep parafoveal and perifoveal VD  in TAO patients  ⏵No significant changes of CC and outer retina flow between groups | No other significant correlations were found | In TAO patients, a significant decrease of RNFLT (especially mean, inferior, and temporal RNFL), as well as an increase in CT and FAZ area, was observed. Despite a marked increase of RPC density in temporal and inferior sectors, superficial VDs of fovea, parafovea and perifovea, in addition to deep VDs of parafovea and perifovea, were significantly reduced in TAO patients compared to controls. |
| 25 | Zhang et al. (67)  2022 | Right eyes of 70 healthy controls and 70 TAO patients (7 subgroups based on 7 grades of NOSPECS classification, 10 patients per group) | A Cirrus HDOCT5000 (Carl Zeiss, Germany) OCTA system, macular scanning using a 6 mm × 6 mm scanning area | Not assessed | Not assessed | ⏵ Reduced FAZ area among TAO patients  ⏵Increased macular and foveal VD and PD in TAO patients | ⏵Negative correlation among TAO severity grade with foveal VD (FVD) and macular PD (MPD)  ⏵Positive correlation of CAS with FVD and FPD | Compared to controls, notable elevation of VD and PD of macular and foveal areas, in addition to FAZ area reduction, was observed among TAO patients. There was a negative correlation between superficial macular flow density and BCVA, which was markedly worse in TAO patients than the controls; altogether, the overall elevation of superficial macular blood flow and its related parameters (FVD, MVD, FPD, and MPD) among TAO patients was accompanied by a declining trend as TAO got advanced. |
| 26 | Xu et al.(68)  2023 | 51 TAO patients classified into active (CAS ≥ 3/7) and stable (CAS ≤ 2/7) groups  39 healthy controls | (SD-OCT) and OCTA images using Nidek RS-3000 Advance device (Nidek, Gamagori, Japan) and a 6 mm × 6 mm scanning mode for evaluation of ONH and fovea | Markedly reduced whole, inferior, and superior radial peripapillary capillary layer (RPCP) densities in active patients compared to other groups and stable patients compared to controls | Non-significantly reduced ONH average PD in the inferior, superior, and whole of the active group compared to stable patients | ⏵ Significantly reduced SRCP PD in active group versus controls (except for foveal, outer temporal, and nasal areas), no significant differences between active and stable groups  ⏵Significantly reduced SRCP PD in stable group versus controls in the fovea, superior inner, outer nasal, and temporal sectors  ⏵ Significantly reduced DRCP PD in the active group compared to other groups and stable patients versus controls (except for foveal and inner temporal regions)  ⏵Increased FAZ area in TAO patients | ⏵Negative correlation of CRT with VF-MD  ⏵Positive correlation of CRT with P100 latency | As for the macular PD (mPD) evaluation, three study groups markedly differed in SRCP in all subfields except for inner temporal, with active TAO having the least PD.  DRCP differed significantly as well in all quadrants among study groups. The evaluation of peripapillary PD, ONH, and RPCP parameters differed notably among all study groups.  Amongst OCTA measurements, DRCP-wPD, followed by RPCP-wPD, FAZ, SRCP-wPD, and ONH-wPD, had the highest ability for distinguishing active and control eyes; altogether DRCP-wPD and RNFL had a high distinguishing value between controls and active TAO patients. |
| 27 | Zeng et al.(69)  2023 | 10 TAO patients (20 eyes) with chorioretinal folds (CRFs)  10 TAO patients (20 eyes) without CRFs (NCRF group) | Prototype AngioVue software 2.0 of the RTVue XR Avanti SD-OCT device (Optovue, Inc., Fremont, CA, USA). A 6 mm × 6 mm fovea-centered scan for evaluation of SLR and DLR VD, RPC density evaluated via HD Angio Disc 4.5 mm mode. | Significantly reduced RPC-wiVD in the CRF group | ⏵Significantly lowered whole-image, peripapillary fields, and temporal fields VD of CRF group | ⏵Significantly reduced SLR in whole image, parafovea, perifovea, and all grid sessions in CRF group  ⏵No significant differences in DLR VDs between the two groups | ⏵Positive correlation of SLR and RPC whole VD with P100 amplitude  ⏵Negative correlation of RFNLT with BCVA, VF-MD, and P100 amplitude  ⏵Positive correlation of RNFLT with VF PSD | Worsening of VF parameters such as BCVA, thicker RNFL, and significant reduction of SLR-mwiVD and RPC-wiVD were observed in the CRF group compared to the NCRF group, indicating a significantly lower VD of whole-image, peripapillary fields, and temporal fields of CRF group, the decreased retinal microvascular density in whom could be an indicator of a sight-threatening condition. |
| 28 | Ozer et al.(70)  2023 | 41 TAO patients (82 eyes); categorized into DON group, ANDON group with CAS≥ 3, and INDON group with CAS< 3  40 healthy controls (40 eyes)  * All DON and ANDON patients underwent 12 weeks of IV MTP treatment* | Avanti RTVue XR instrument (OptovueInc, Fremont, Calif.), a 4.5 ×4.5 mm rectangular area for optic disc scan | Reduced total, hemi-superior and hemi-inferior RPC VD in the DON group (no significant differences in these measures after IV MTP therapy) | Reduced peripapillary VD in DON patients compared to other groups | Not assessed | ⏵Correlation of IOP with lamina cribrosa thickness  ⏵Correlation of RNFLT with VF mean defect | Despite no significant differences in total, superiorhemi and inferiorhemi-sectors RNFLT among groups, peripapillary VD was reduced in DON patients. Following IV MTP therapy, an increase of BCVA and a decrease of TSHR Ab, as well as the superiorhemi-sector RNFLT, IOP, and proptosis, were observed. Furthermore, compared to other groups, a decrease in total, hemi-superior and hemi-inferior RPC VD was observed in the DON group. After IV MTP therapy, there were no notable differences in total, hemi-superior, and hemi-inferior RPC VD. |
| 29 | Lee et al.(71)  2023 | 57 active TAO patients undergoing 12 weeks of systemic IV glucocorticoid (GC) therapy | SS-OCT (DRI OCT Triton, Topcon, Tokyo, Japan), 3.0 × 3.0-mm en face images of the macula obtained by OCT-A | Not assessed | Not assessed | ⏵ Increased sFAZ and decreased dFAZ following corticosteroid therapy  ⏵Increased superficial and deep capillary plexus density (sCPD and dCPD, respectively) by initiation of corticosteroid therapy (later having a declining trend) | ⏵Confirmed correlation between serum thyroid-stimulating immunoglobulin (TSI) and TSH receptor (TSHR) antibody levels and improvement of chorioretinal capillary perfusion, muscle thickness  and clinical symptoms  ⏵Negative correlation of TSHR Ab with sFAZ and dFAZ at 2 months of IV GC therapy | As a result of GC treatment, chorioretinal blood flow, CAS, serum autoantibody levels, and extraocular muscle thickness were improved.  As of the evaluation of chorioretinal vascular structures, including the sCPD, dCPD, and FAZ area, after two months of treatment, increased sFAZ, and decreased dFAZ were observed compared to the baseline.  The serum TSI and TSHR Ab levels had a continuous decreasing trend accompanied by attenuation of CAS until two months of follow-up, suggesting that the reduction in levels of TSI and TSHR Ab can be a prognostic factor of restoration of chorioretinal capillary perfusion and improvement of clinical symptoms after IV GC therapy of TAO patients. |
| 30 | Tu et al.(72)  2023 | 24 healthy controls  81 TAO patients (50 NDON and 31 DON patients) | Optovue RTVue XR Avanti; Optovue, Inc., Fremont, CA,  USA, using 3 × 3 mm2 foveal scans | Not assessed | Not assessed | ⏵Significantly reduced SRCP density in TAO patients compared to controls  ⏵Significantly reduced SRCP density in DON compared to NDON group  ⏵No significant changes in DRCP between the TAO group and controls | ⏵Correlation of ganglion cell complex layer (GCCL) thickness and inner retinal microvascular density in TAO patients  ⏵Correlation of GCCL thickness with SRCP and DRCP density | Subsequent to the measurement of contrast sensitivity function (CSF), significant attenuation of SRCP density was observed in all TAO patients compared to controls (no substantial changes in DRCP), in addition to a decrease of GCCL thickness in DON compared to other groups; altogether indicating alterations of CSF in early stages of DON which is associated with SRCP density and can be prognostic of visual disruption. |
| 31 | Abrishami et al.(73)  2023 | 30 healthy controls  55 TAO patients (32 patients with CAS < 3 (inactive TAO) and 23 patients with CAS ≥ 3 (active TAO) at first examination) | AngioVue RTVue XR, Avanti, Optovue, Fremont, CA, USA, software version: 2018,0,0,18 with a scan size of 6 mm × 6 mm for assessment of Superficial and deep capillary plexus  AngioVue RTVue XR Avanti, Optovue, Fremont, CA, USA for the analysis of ONH neuro-vasculature using a 4.5 × 4.5 mm ONH centered scan | Significantly reduced wi-RPC density and inferior hemisphere wi-RPC density in TAO patients, but no significant differences between active and inactive TAO groups | Not assessed | ⏵No significant changes of foveal superficial and deep VDs (SVD and DVD) between patients and controls, in addition to no significant differences between two subgroups of TAO  ⏵Significantly enlarged FAZ area in TAO patients compared to controls, in addition to significantly greater FAZ area in active patients compared to inactive patients  ⏵No significant differences of parafoveal SVD and DVD between patients and controls, except for a significant reduction of parafoveal SVD-superior in TAO patients  ⏵No significant differences of parafoveal SVD and DVD between active and inactive patients, except for significant reduction of parafoveal DVD, parafoveal DVD-superior and inferior in active TAO patients | No other significant correlations were found | FAZ can be considered as a prognostic parameter of TAO activity and vasculopathy. As for the parameters of peripapillary flow and RNFLT, TAO patients had lower RNFLT and a remarkably decreased RPC density compared to controls; however, there was no significant difference in parameters of peripapillary flow and RNFLT between active and inactive TAO patients. |
| 32 | Jamshidian Tehrani et al.(74)  2023 | 18 patients (25 eyes) with NANC TAO (candidates of cosmetic decompression surgery) with no cases of active TAO or DON according to CAS evaluation | RTVue Avanti SD-OCT system with the AngioVue software (version 2016.1.0.26; Optovue, Inc., Fremont, CA. A 3 × 3 mm macular scan and a 4.5 × 4.5 mm optic disc-centered scan for evaluation of ONH | Significantly elevated postoperative peripapillary SV density of RPC, No significant changes in small vessels (SV) and all vessels (AV) density measures of whole image, inside disc, superior and inferior Hemifield areas | Significantly increased Cup/Disc horizontal ratio postoperatively | ⏵Significantly reduced whole image, foveal and superior-Hemi superficial retinal VD postoperatively  ⏵No significant alterations of deep retinal VD parameters postoperatively  ⏵Significantly reduced foveal and superior-Hemi superficial CC VD postoperatively | Correlation of TAO-associated VD decline with disease activity more than orbitopathy stage | After a three-month follow-up, a significant increase of mean RPC of small peripapillary vessels and reduction of peripapillary and Inferior-Hemifield area mean RNFL thickness was observed after surgery in addition to notable attenuation of superior-Hemifield and fovea choriocapillaris. Furthermore, following surgery, a reduction of peripapillary and inferior hemifield RNFL thickness and an inclined cup/disc horizontal ratio were observed. |
| 33 | Dogan et al.(75)  2023 | 40 healthy controls  78 TAO patients (156 eyes)  (54 stable patients with CAS<4 and 24 active TAO patients with CAS ≥ 4)  patients were also divided into mild, moderate to severe, and sight-threatening groups according to EUGOGO classification | Topcon swept source OCT/OCT angiography (OCTA) DRI OCT Triton (Topcon Co. Japan), 3 × 3 mm scans centered on the fovea | Not assessed | Not assessed | ⏵Increased mean and central DVD (m-DVD and c-DVD) in active TAO compared to inactive patients  ⏵Increased s-FAZ and d-FAZ, reduced mean and central superficial vessel density (m-SVD and c-SVD) in patients with high TRAB  ⏵Reduced m-SVD in high TPO patients | No other significant correlations were found | Elevated mean and central DVD (m-DVD and c-DVD) were observed in active TAO patients with no notably different SFCT among active and inactive groups. In patients with high thyroid-stimulating hormone-receptor autoantibodies (TRAB), increased s-FAZ and d-FAZ were observed in addition to reduced mean and central superficial vessel density (m-SVD and c-SVD). Patients with elevated levels of human thyroglobulin (hTG) and TRAB had increased SFCT thickness. Patients with heightened TPO levels only had reduced m-SVD. No significant difference was found among subgroups of the EUGOGO classification. |
| 34 | Ceylanoglu et al.(76)  2023 | 18 children with mild and inactive GO (36 eyes)  20 age-matched controls (40 eyes) | XR Avanti AngioVue OCT-A (Optovue, Fremont, California, USA) (Version 2017.1.0.151), A 6 × 6 mm macular scan and a 4.5 × 4.5 mm rectangular scan centered on the optic disc | Not assessed | No significant changes of peripapillary vascular parameters | No significant changes in FAZ area, acircularity index, macular SCP, and DCP | No other significant correlations were found | Despite reduced inferior temporal RNFL thickness in GO children, there were no significant differences in ON thickness, peripapillary, and macular vascular parameters, including FAZ area, acircularity index, macular SCP, and DCP. |
| 35 | Sener et al. (77)  2023 | 40 controls  41 TED patients (divided into 22 inactive TED (ITED) patients (CAS<3) and 19 active TED (ATED) patients (CAS ≥ 3) who were subdivided into DON and NDON patients)  *Only one eye of the subjects was included. | Avanti RTVue XR instrument (Avanti RTVue XR; Optovue, Fremont,  CA, USA), a 4.5 × 4.5-mm scan for evaluation of optic disc | ⏵Significantly decreased annular and hemifield (superior and inferior) RPC density was observed in the ATED group compared to the controls and ITED⏵Significantly decreased RPC density of DON group. | ⏵Significantly decreased peripapillary, hemisuperior, and hemiinferior density in DON compared to NDON patients | Not assessed | No other significant correlations were found | Decrease in TSHr Ab level correlated with RPC density, and the elevation of pRNFL and parapapillary choroidal microvasculature (PPCMv) density was observed in active TED patients. The correlation of CAS with PPCMv density and RPC density was confirmed as positive and negative, respectively.  Despite unchanged pRNFL thickness and PPCMv density measures in the DON group, they had markedly attenuated RPC density. Altogether, changes in peripapillary microvascular perfusion, which intensify with the advancement of CAS, may be a leading cause of DON. |
| 36 | Sun et al.(78)  2024 | 26 active TAO patients with CAS ≥3  25 inactive TAO patients with CAS<3 | A single OCTA system (Spectralis OCT, Heidelberg Engineering,  Heidelberg, Germany) a 3.0 × 3.0 mm fovea-centered scan | Not assessed | Not assessed | No significant changes in SVD and DVD between groups | No other significant correlations were found | A simultaneous increase of choroidal vascularization and retrobulbar blood flow was observed in active TAO patients. Despite a significant increase of CT and CVI in active TAO patients, retinal OCTA parameters, including SVD and DVD, showed no significant decrease between the two groups |
| 37 | Fan et al.(79)  2024 | 18 DON patients (29 eyes) undergoing high-dose IV MTP therapy  16 (32 eyes) healthy controls | Prototype AngioVue software 2.0 of the RTVue XR Avanti spectral domain OCT device (Optovue, Inc., Fremont, CA, USA) | ⏵Significantly reduced whole image and peripapillary RPC VD in pretreatment DON group compared to controls (no significant changes of these parameters after treatment)  ⏵ No significant differences of inside disc RPC VD between DON and control group, and, DON patients before and after treatment | Not assessed | ⏵Notable decline of macular VD in DON patients compared to controls (significant reduction of SLR and DLR mwiVD  ⏵Reversal of declining trend of macular VD in patients in addition to VF improvement, elevation of SLR-mwiVD and DLR-mwiVD subsequent to therapy | Notable correlation of SLR and DLR mwiVD elevation wth BCVA, VF-MD and VF-PSD improvement susequent to therapy | The significant improvement of parameters related to macular VD subsequent to high dose IV MTP treatment indicates the effectiveness of this treatment option in DON. |
| 38 | Wu et al.(80)  2022 | 50 subjects (98 eyes) in total, comprising of 34 normal eyes, 64 TAO eyes (39 DON and 25 NDON eyes)  *11 DON patients received V steroid therapy, 5 unresponsive to corticosteroid therapy DON patients underwent surgical decompression | The RTVue-XR Avanti (Opto Vue, Inc, Fremnt, CA,  USA), using a 6×6 mm2 field size for retinal scanning | ⏵Significantly reduced RPC density in all segments except for inside disc, superior, inferior and nasal areas in TAO patients compared to controls, and DON compared to NDON patients  ⏵No significant changes of whole, peripapillary, inside disc RPC densities in DON patients after surgical and medical treatment (despite improvement of visual field and acuity) | Not assessed | Not assessed | Significant correlation of whole choroidal RPC density reduction with VF defect of DON patients (not in normal and NDON subjects) | Despite the notable improvement of VF subsequent to treatment in DON patients, insignificant changes of choroidal RPC density measures immidiatey after surgery, indicates the potential role of choridal RPC as diagnostic parameter of early stages of DON. |
| 39 | Wu et al.(81)  2019 | 125 subjects (93 GO patients and 32 normal controls) | RTVue XR Avanti Spectral Domain OCT system (Optovue, Inc., Fremont, CA, USA), a 3 × 3 mm fovea centered area | Not assessed | Not assessed | Significantly reduced RCD of superior, nasal and total annular zone (TAZ) in SRCP and DRCP of GO patients compared to controls | ⏵Significant correlation of RCDsand CAS score values  ⏵Important correlation of visual acuity with reduction of RCD | The reduced measures of RCDs in GO patients were mostly relevant to disease activity status and serum antibodidies; however, signifcantly higher measures of IOP and proptosis was also observed in GO patients. |
| 40 | Bouazzaoui et al.(82)  2021 | 10 inactive GO patients (20 eyes)  10 age-matched healthy controls (20 eyes) | Optovue RTVue XR Avanti, AngioVue, for obtaining 6 × 6 mm macular scans | Not assessed | Not assessed | ⏵Significantly decreased SCP-VD in GO patients compared to controls  ⏵No significant changes of FAZ area and DCP-VD in GO patients compared to controls | No other significant correlations found | OCTA can be applied as novel imaging modality for monitoring of GO patients through evaluation of changes of retinal vascular structures. |
| 41 | Fang et al.(83)  2024 | 44 inactive TAO patients (65 eyes) with a CAS<3 | Optovue Inc., Fremont, CA, USA; a 3 × 3mm scanning area around the fovea for evaluation of DCP and SCP-VD, a 4.5 × 4.5 mm area around the optic disk for peripapillary VD assessment | Not assessed | ⏵Significant correlation of whole peripapillary VD and WEM  ⏵Notable correlation of WEM with VDs of inferior-nasal, inferior-temporal, superior-temporal and superior-nasal peripapillary areas | Significant correlation of SCP-VDs of whole parafoveal, superior, inferior, nasal and temporal parafoveal regions with WEM after adjustment by age, gender, AL and IOP. (no significant correlation of WEM with DCP-VDs of aforementioned areas) | No other significant correlations found | In this study the correlation of retinal VD and whole eye movement (WEM), as a reliable parameter of IOP or orbital compliance assessment, was evaluated; the results of which indicated an important association between reduction of retinal VD and stiffness of orbit in inactive TAO patients |
| 42 | Zeng et al.(84)  2023 | 20 TAO patients with CRFs (35 eyes); CRF patients divided into groups of CRF with optic disc edema (ODE) (18 eyes) and non-ODE CRF patients (17 eyes)  20 normal controls (40 eyes) | Prototype AngioVue software 2.0 of the RTVue XR Avanti spectral domain OCT device (Optovue, Inc., Fremont, CA, USA), a 6 mm × 6 mm scanning area of scan centered on fovea | Not assessed | Not assessed | ⏵Significantly declined whole image and perifoveal VDs of SLR and DLR in TAO patients with CRF compared to normal subjects. (no significant differences between CRF patients with or without ODE)  ⏵Notably reduced parafoveal SLR VD in CRF patients compared to controls  ⏵No remarkable differences of foveal SLR and DLR VDs among groups  ⏵Significantly reduced whole image, foveal and perifoveal CC-VD in TAO patients with CRFs compared to normal subjects, and in non-ODE patients compared to CRF group with ODE | ⏵Negative correlation of all VD parameters with BCVA, VF-PSD, and P100  latency  ⏵Positive correlation of all VD parameters with VF-MD and P100 amplitude | The notable VD decline in macular CC and retina among TAO patients with CRFs was associated with visual impairment. Additionally, these changes of VD were significant while comparing macular CC of CRF patients with or without ODE. |
